# Supplementary material for: Complexity and critical thresholds in the dynamics of visceral leishmaniasis
Source: R Soc Open Sci. 2020 Dec 16;7(12):200904. doi: 10.1098/rsos.200904 (PMC7813240; doi:10.1098/rsos.200904)
Supplement: Understanding R0, biting heterogeneity, backward bifrucation and system resilinece. [file rsos200904supp1.docx]

## **Appendix**.

(See main text titled *“Complexity and critical thresholds in the dynamics of Visceral Leishmaniasis”*, S. Bilal, R. C. Rivera, A. Mubayi, and E. Michael).

The supporting information contains the following:

1. General representation of compartmental models
2. Negative binomial transmission term
3. The next generation matrix approach to calculate $R_{0}$
4. Coefficients of the sextic polynomial and simpler explanation for backward bifurcation
5. Integral Stability for measuring resilience
6. System sensisitivity using PRCC and tornado plot
7. Expression for $R_{0}$ in two patch model

### General representation of compartmental models

A typical compartmental model divides the host and vector populations into separate classes such as susceptible, exposed, infectives, and recovered individuals ^1, 2^ and describes the progression between these classes. In most vector borne diseases the vectors typically are not able to recover from infection thus there is no recovered class for vectors. During an emerging outbreak, all individuals of host populations are susceptible; once infected they can remain dormant (exposed) for some time before becoming infectious or they immediately become infectives and can infect biting vectors. Infected individuals may (1) recover, (2) may die due to infection (3), may develop some other nonfatal complications, or (3) become susceptible again.

In general, in this system, a heterogeneous population (including vectors) can thus be grouped into *N* homogeneous compartments. We can rearrange the compartments such that the first *m* of them correspond to the infected (including symptomatic and asymptomatic) while the rest correspond to uninfected classes. The time evolution of the state of the population ${\{x}_{i}\}$ has the general form:

|  | $\frac{dx_{i}}{dt}=\mathcal{F}_{i}-\mathcal{V}_{i},$ | (1) |
| --- | --- | --- |

where $\mathcal{F}_{i}$ represents input rate of newly infected individuals in the $i-th$ compartment and $\mathcal{V}_{i}=\mathcal{V}_{i}^{-}-\mathcal{V}_{i}^{+}$ with $\mathcal{V}_{i}^{\pm}$ is the transfer in/out of the compartment $i$ by all other means. Writing a compartmental model in the form of Eq. 2 facilitates the calculation of the basic reproductive ratio $R_{0}$ for complex models involving multiple hosts or vectors ^3^ as shown in the Appendix. This framework also allows development of a hierarchical set of specific models of increasing or decreasing complexity.

### The next generation matrix approach for $\boldsymbol{R}_{\boldsymbol{0}}$

The number of secondary infections as a result of a single infectious host/vector ($R_{0}$) is given by the spectral radius of the next generation matrix ^3^ ($FV^{-1})$, where elements of $F$ and $V$ are given by

|  | $F_{ij}=\frac{\partial\mathcal{F}_{i}}{\partial x_{j}}$ $V_{ij}=\frac{\partial\mathcal{V}_{i}}{\partial x_{j}}$ |  |
| --- | --- | --- |

with $i,j=1,\ldots,m$ and each matrix is evaluated at the disease free equilibrium (DFE). The matrices $F$ and $V$ for full model (see main text) are obtained from Eq. (2) and (6) (see main text) by noting that it has eight infected compartments ($E_{H},I_{H},T_{H},K_{H},{I_{A},I_{B},E}_{v},I_{v}$):

|  | $\begin{aligned} F=\left[ \begin{matrix} 0 & 0 & 0 & 0 & 0 & 0 & 0 & \frac{b{\alpha_{H}\beta}_{H}S_{H}^{0}}{N_{av}^{0}} \\ 0 & 0 & 0 & 0 & 0 & 0 & 0 & 0 \\ 0 & 0 & 0 & 0 & 0 & 0 & 0 & 0 \\ 0 & 0 & 0 & 0 & 0 & 0 & 0 & 0 \\ 0 & 0 & 0 & 0 & 0 & 0 & 0 & \frac{b{\alpha_{A}\beta}_{A}S_{A}^{0}}{N_{av}^{0}} \\ 0 & 0 & 0 & 0 & 0 & 0 & 0 & \frac{b{\alpha_{B}\beta}_{B}S_{B}^{0}}{N_{av}^{0}} \\ \frac{b{\alpha_{H}\beta}_{H}S_{H}^{0}}{N_{av}^{0}} & \frac{b{\alpha_{H}\beta}_{H}S_{H}^{0}}{N_{av}^{0}} & 0 & \frac{b{\alpha_{H}\beta}_{H}S_{H}^{0}}{N_{av}^{0}} & \frac{b{\alpha_{H}\beta}_{H}S_{H}^{0}}{N_{av}^{0}} & \frac{b{\alpha_{H}\beta}_{H}S_{H}^{0}}{N_{av}^{0}} & 0 & 0 \\ 0 & 0 & 0 & 0 & 0 & 0 & 0 & 0 \end{matrix} \right] \\ V=\left[ \begin{matrix} Q_{1} & 0 & 0 & 0 & 0 & 0 & 0 & 0 \\ -f\sigma_{1} & Q_{2} & 0 & 0 & 0 & 0 & 0 & 0 \\ 0 & -\gamma_{1} & Q_{3} & 0 & 0 & 0 & 0 & 0 \\ 0 & 0 & {-\eta r}_{T} & Q_{4} & 0 & 0 & 0 & 0 \\ 0 & 0 & 0 & 0 & Q_{6} & 0 & 0 & 0 \\ 0 & 0 & 0 & 0 & 0 & Q_{7} & 0 & 0 \\ 0 & 0 & 0 & 0 & 0 & 0 & Q_{v} & 0 \\ 0 & 0 & 0 & 0 & 0 & 0 & -\sigma_{v} & \mu_{v} \end{matrix} \right] \end{aligned}$ | (2) |
| --- | --- | --- |

The spectral radius of the next generation matrix $FV^{-1}$is found using Mathematica software:

|  | $\begin{matrix} {R_{0}}^{2}= \left( \frac{b^{2}\beta_{v}\sigma_{v}N_{v}^{0}}{\mu_{v}Q_{v}N_{tot}^{0}} \right)\sum_{l=\{H,A,B\}} \frac{\beta_{l}\alpha_{l}m_{l}N_{l}^{0}}{N_{tot}^{0}} \\ m_{H}=\frac{\alpha_{H}C_{H}\left( Q_{3}Q_{4}\left( f\sigma_{H}+\rho_{1}Q_{2} \right)+\rho_{2}\eta r_{T}\gamma_{H}f\sigma_{H} \right)}{Q_{1}Q_{2}Q_{3}Q_{4}} \\ m_{A}=\frac{\alpha_{A}C_{A}}{Q_{6}} \\ m_{B}=\frac{\alpha_{B}C_{B}}{Q_{7}} \\ N_{tot}^{0}=\sum_{l=\{H,A,B\}} \alpha_{l}N_{l}^{0}, \end{matrix}$ | (3) |
| --- | --- | --- |

### Negative binomial transmission

We adopt the formalism developed for modeling contact heterogeneity in directly transmitted diseases and dengue ^4, 5^ and extend it to multi-host VL model. Assuming that the number of VL transmission causing bites on *m^th^* individual host are Poisson distributed with mean $\theta_{m}$. Further, Poisson means themselves are random variables with a Gamma distribution in the population (a reasonable assumption given that mean bites have to be greater than zero and can take large range of values) with shape parameter $k_{j}$ and a rate parameter $s_{c}$ (scale parameter $1/s_{c}$):

|  | $g\left( \theta\right)=\frac{s_{c}}{{\Gamma(k}_{j})}\theta^{k_{j}-1}e^{-s_{c}\theta}$ | (4) |
| --- | --- | --- |

Then the conditional distribution of effective number of bites (bites sufficient to transmit the diseases) $X_{i}$ is given by:

|  | $P\left( X_{i}=x \right\vert\theta_{m}=\theta)=\frac{e^{-\theta}\theta^{x}}{x!}$ | (5) |
| --- | --- | --- |

Consequently, the marginal probability distribution of effective risky bites is obtained by integrating Eq. (3) over the entire range of $\theta$ given against $g\left( \theta\right)$:

|  | $\begin{matrix} P\left( X_{i}=x \right)=\int_{0}^{\infty} \frac{s_{c}}{{\Gamma(k}_{j})}\theta^{k_{j}-1}e^{-s_{c}\theta}\frac{e^{-\theta}\theta^{x}}{x!}d\theta\\ P\left( X_{i}=x \right)=\left( \begin{matrix} {x+k}_{j}-1 \\ x \end{matrix} \right)\left( \frac{1}{1+s_{c}} \right)^{x}\left( 1-\frac{1}{1+s_{c}} \right)^{k_{j}},x=0,1,2\ldots\end{matrix}$ | (6) |
| --- | --- | --- |

Eq. (6) shows that the effective bites follow a negative binomial distribution with mean $\frac{k_{j}}{s_{c}}$ . The mean effective bites on hosts should be

|  | $\begin{matrix} \frac{k_{j}}{s_{c}}=\frac{b\beta_{l}\alpha_{l}}{\sum_{l} \alpha_{l}N_{lj}}I_{vi} \\ \left( l=H Human, l=A,B for reservoirs \right) \end{matrix},$ | (7) |
| --- | --- | --- |

where $\frac{b\beta_{l}\alpha_{l}}{\sum_{l} \alpha_{l}N_{lj}}I_{vi}$ is the homogeneous mixing effective bites. Then probability that a susceptible host escapes infection is ${p=\left( 1+\frac{b\beta_{l}\alpha_{l}}{k_{j}\sum_{l} \alpha_{l}N_{lj}}I_{vi} \right)}^{{-k}_{j}}$ (put $x=0$ in Eq. 6 and use $\frac{k_{j}}{s_{c}}=\frac{b\beta_{l}\alpha_{l}}{\sum_{l} \alpha_{l}N_{lj}}I_{vi}$ ) or becomes infected is $1-p$. In the absence of any demographic parameters the susceptibles become infected in discrete steps with the risk of susceptibles becoming infectious $(1-p)$. The relationship between risk and rate^6^ $risk=1-e^{rate}$, gives the rate of infection of susceptible hosts as

|  | $\lambda_{lj}=k_{i}\log\left( 1+\frac{b\beta_{l}\alpha_{l}}{k_{j}\sum_{l} \alpha_{l}N_{lj}}I_{vi} \right).$ | (8) |
| --- | --- | --- |

Through a similar argument on probability of effective bites on infectious hosts by a susceptible sandfly vector, the rate of infection for susceptible sandfly population is

|  | $\begin{matrix} \lambda_{vj}=k_{j}\log\left( 1+\frac{b\beta_{v}\left[ \alpha_{H}C_{H}\left( I_{Hj}+\rho_{1j}E_{Hj}+\rho_{2j}K_{Hj} \right)+\alpha_{A}C_{A}I_{Aj}+\alpha_{B}C_{B}I_{Bj} \right]}{k_{j}\sum_{l} \alpha_{l}N_{lj}} \right) \\ \frac{k_{j}}{s_{c}}=\frac{b\beta_{v}\left[ \alpha_{1}C_{H}\left( I_{Hj}+\rho_{1j}E_{Hj}+\rho_{2j}K_{Hj} \right)+\alpha_{A}C_{A}I_{Aj}+\alpha_{B}C_{B}I_{Bj} \right]}{\sum_{l} \alpha_{l}N_{lj}} \end{matrix}$ | (9) |
| --- | --- | --- |

Where $k_{j}$ and the rate parameter $s_{c}$ (scale parameter $1/s_{c}$) correspond to the Gamma distribution of mean number of effective bites (cf Eq. 4-5). In these expressions $k_{j}$ characterizes the level of heterogeneity of bites (susceptible humans/reservoirs by infectious sandfly vectors and infectious humans/reservoirs by susceptible vectors) ^4, 7^ and is assumed to be same for all hosts (humans and reservoirs A and B) and in all patches. Finally, incorporating movement of humans between *n*-patches we obtain:

|  | $\begin{matrix} \lambda_{lj}=\sum_{i=1}^{n} k_{i}\log\left( 1+\frac{b\beta_{l}\alpha_{l}p_{ij}}{k_{i}\sum_{l} \alpha_{l}N_{li}}I_{vi} \right),l=H \\ \lambda_{lj}=\sum_{i=1}^{n} k_{i}\log\left( 1+\frac{b\beta_{l}\alpha_{l}\Delta_{ij}}{k_{i}\sum_{l} \alpha_{l}N_{li}}I_{vi} \right), l=A,B \\ \lambda_{vj}=\sum_{i=1}^{n} k_{i}\log\left( 1+\frac{b\beta_{v}\left[ \alpha_{H}C_{H}p_{ji}\left( I_{Hi}+\rho_{1i}E_{Hi}+\rho_{2i}K_{Hi} \right)+\Delta_{ji}\alpha_{A}C_{A}I_{Ai}+\alpha_{B}\Delta_{ji}C_{B}I_{Bi} \right]}{k_{i}\sum_{l} \alpha_{l}N_{li}} \right) \\ \Delta_{ij}=\left\{ \begin{aligned} 1 i=j \\ 0 i\neq j \end{aligned} \right., \end{matrix}$ | (10) |
| --- | --- | --- |

Where $p_{ij}$ are elements of residence times matrix for humans-time spent by humans of patch *j* in *i* and $\Delta_{ij}$ are elements of residence times matrix for reservoirs A and B. Since our model assumes that non-human reservoirs do not move between patches, in that case $\Delta_{ij}$ is an identity matrix.

### Coefficients of polynomials

To calculate the coefficients of the sextic polynomial equation for the human force of infection the following procedure is used

1. Write down the expressions for force infection for human, two reservoirs, and sand flies
2. Divide both sides of each of these expression by respective force of infection ${\{\lambda}_{i}\}$, $\lambda_{v}$.
3. Add all the resulting expressions
4. Express all the $\lambda_{A}$,$\lambda_{B}$, $\lambda_{v}$ in terms of $\lambda_{H}$ using the relation $\lambda_{A}=\frac{\alpha_{A}}{\alpha_{H}}\lambda_{H}, \lambda_{B}=\frac{\alpha_{B}}{\alpha_{H}}\lambda_{H}$.
5. Identify the coefficients of ${\lambda_{H}}^{n}$,$n=(0, 1, 2, 3, 4, 5, 6)$

When only humans and sand fly vectors are considered , the polynomial is quadratic and has coefficients $A_{2},A_{1},A_{0}$ given by the following:

|  | $\begin{matrix} A_{2}=\left( F_{2}^{H} \right)^{2}+\frac{b\beta_{v}F_{2}^{H}\left[ Q_{3}Q_{4}\left( f\sigma_{H}+\rho_{1}Q_{2} \right)+\rho_{2}\eta r_{T}\gamma_{H}f\sigma_{H} \right]}{\mu_{v}Q_{v}Q_{1}Q_{2}Q_{3}Q_{4}} \\ A_{1}=2F_{2}^{H}+\frac{b\beta_{v}\left[ Q_{3}Q_{4}\left( f\sigma_{H}+\rho_{1}Q_{2} \right)+\rho_{2}\eta r_{T}\gamma_{H}f\sigma_{H} \right]}{\mu_{v}Q_{v}Q_{1}Q_{2}Q_{3}Q_{4}} \\ A_{0}=1-\left( R_{0} \right)^{2} \\ F_{2}^{H}=\frac{1}{Q_{1}}+\frac{f\sigma_{H}}{Q_{1}Q_{2}}+F_{1}^{H} \end{matrix}-\frac{\left( R_{0} \right)^{2}(1-\delta_{H}F_{1}^{H})}{\mu_{H}}$ | (11) |
| --- | --- | --- |

Using the condition ${A_{1}}^{2}-4A_{2}A_{0}=0$ one can obtain the critical threshold $R_{c}$. Similar calculation for full model is analytically not possible therefore we solved the full sextic polynomial numerically and obtained the threshold $R_{c}$.

### Understanding the backward bifurcation phenomenon via a simpler example

In the simplest case of a SI-SI (SI for humans and SI for sandflies) model for VL transmission, it is easy to show ^8^ (using the criterion ${A_{1}}^{2}-4A_{2}A_{0}=0$ ) that in the presence of disease induced death in humans backward bifurcation occurs under the following conditions ^8^:

|  | $\begin{matrix} d_{H}>\mu_{H} \\ \frac{d_{H}}{\mu_{H}}>1+\frac{b\beta_{v}}{\mu_{v}} \\ \mu_{v}>\frac{b\beta_{v}}{\frac{d_{H}}{\mu_{H}}-1} \end{matrix}$ | (12) |
| --- | --- | --- |

The second of these inequalities suggests that the rate of transmission from humans to sandflies, places a constraint on sandfly and human demographic parameters (diseased induced death and natural mortality) to observe backward bifurcation. This effect is nonlinear, and shifts the balance of excessive transmission to sandflies thereby leading to an stable endemic state even when $\boldsymbol{R}_{\boldsymbol{0}}\boldsymbol{<1}$. The shift in excessive transmission to sandflies in VL is similar to the TB model ^9^ where transmission from explicit re-infection causes backward bifurcation. Similar to TB, in our model the increase in human infection prevalence occurs, however, there are two points to note: 1) new infections are generated by transmission from other population (sandfly population) even though disease induced death in humans acts like a leakage in the system, 2) increment in infection does not occur in human but in sandfly population which then feeds back into human population.

### System resilience: Integral Stability

Integral stability ${0\leq d}_{IS}\leq1$ is a measure of ecological resilience of a state combining the two aspects of resilience ^10^, namely

1. Resistance ($R$)
2. Precariousness ($Pr$).

To define these two terms, we recall the definition of basin of attraction of any stable state (DFE/endemic state): it is the set of all perturbations with-in a volume $Y_{i}$ which eventually lead to the underlying attractor *i*. Resistance is the capacity of overcoming changes following a perturbation-this can be calculated from the Lyapunov exponents using the boundary as the initial condition. Lyapunov exponents capture the stability and it’s strength of an underlying attractor, using an initial condition within and up to the boundary of basin of attractionPrecariousness measures the current distance from a trajectory to its threshold limit of perturbation, which if crossed the trajectory would never recover (i.e. perturbation lies outside the basin of attraction). Precariousness of an attractor ($Y_{i}$) is measured as the minimum perturbation required to be outside its basin of attraction. Mathematically the definition of Resistance, Precariousness, and integral stability are as follows:

|  | $\Pr\left( Y_{i} \right)=inf\left\{ dist\left( a,b \right)\vert a\in Y_{i},b\in\Delta M(Y_{i}) \right\}$ | (13) |
| --- | --- | --- |

where $\boldsymbol{\Delta M(}\boldsymbol{Y}_{\boldsymbol{i}})$ is the boundary of the basin of attraction $\boldsymbol{M(}\boldsymbol{Y}_{\boldsymbol{i}})$ of the attractor $\boldsymbol{(}\boldsymbol{Y}_{\boldsymbol{i}})$. The largest local Lyapunov exponent (calculated using a particular initial condition ($y)$) is related to the resistance at that initial point.

|  | $R\left( y \right)=-max\{\Lambda_{1},\Lambda_{2},\cdots\Lambda_{dim}\}$ | (14) |
| --- | --- | --- |

Then the definition of integral stability $d_{IS}(Y_{i})$ of the attractor $Y_{i}$ is as follows:

|  | $d_{IS}(Y_{i})=\frac{Pr(Y_{i})\int_{y\in M(Y_{i})} R(y)\delta(y)}{\sum_{j} Pr(Y_{j})\int_{y\in M(Y_{j})} R(y)\delta(y)}$ | (15) |
| --- | --- | --- |

The integral is performed over the basin of attraction volume element $\boldsymbol{\delta(y)}$ which is related to ‘Latitude’. The exact values of precariousness, resistance, and latitude are not important, giving us only ${0\leq d}_{IS}(Y_{i})\leq1$ which can be compared to other attractors of the system ^10^. Thus when only DFE (endemic equilibrium) exists then the value of $d_{IS}=1$, in the region where it coexists with the endemic equilibrium (DFE) then it gradually decreases and becomes zero when only endemic equilibrium (DFE) exists. Therefore, in a multihost system the changing backward bifurcation regions indicated a change in resilience of the system as a function of human/reservoir populations-see text for demonstration.

### System sensitivity: PRCC

We employ PRCC techniques for the global sensitivity analysis ^11^ to investigate sensitivity to system parameters. The distribution of parameters, together with mean and standard deviation, for the analysis are shown in Figure 1S. PRCC for sandfly threshold $(N_{vc})$ and human infection threshold ${(I}_{c})$ are presented in the text. The corresponding PRCC for threshold reproduction $R_{c}$ is shown in Figure 2S.


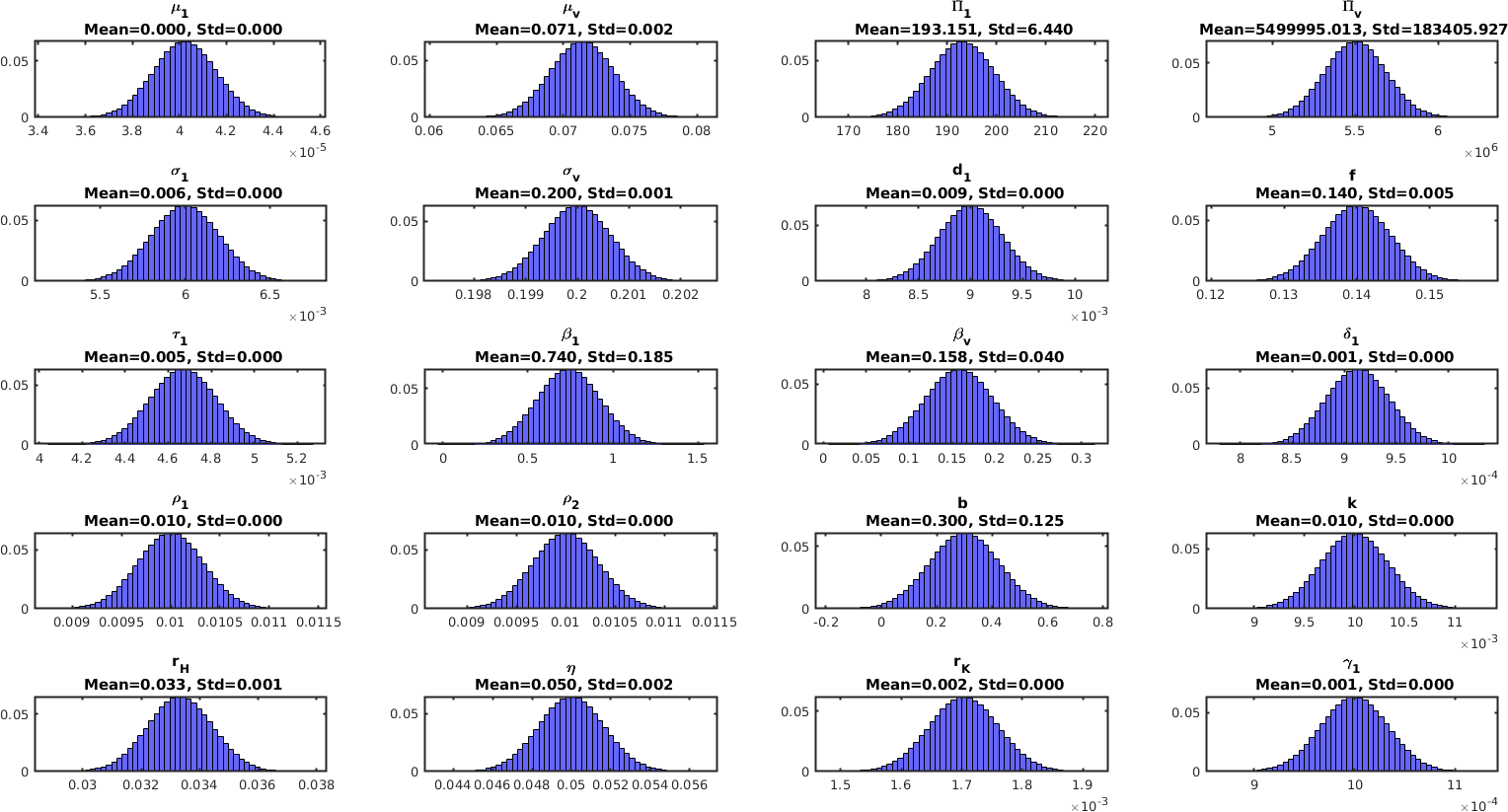


Figure 1S. The distribution of parameters considered to evaluate PRCC of the thresholds ${(N}_{vc},I_{c},R_{c})$. Each subplot shows the mean and standard deviation of the distributions.


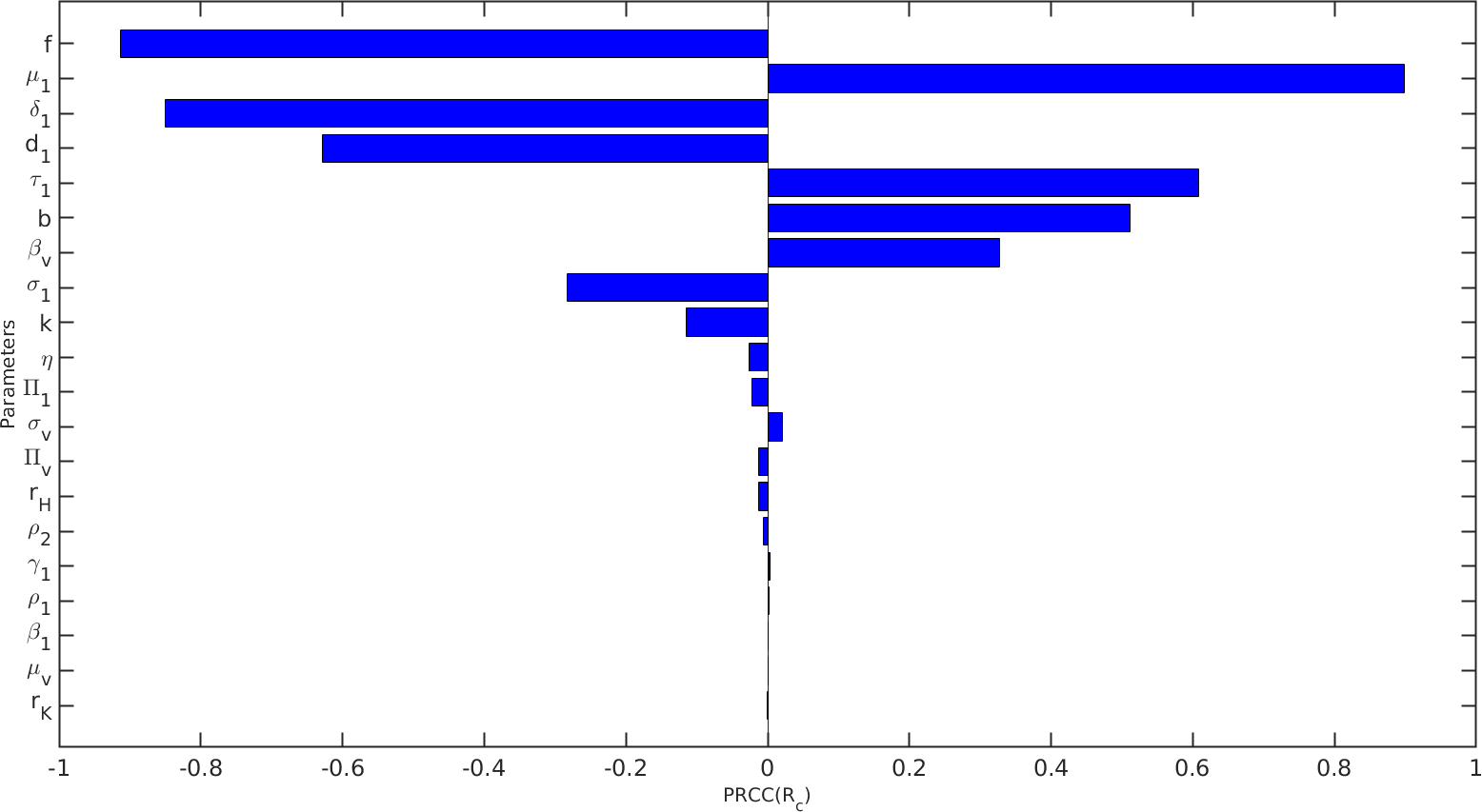


Figure 2S. A tornado plot showing sensitivity of $R_{c}$ $\left( PRCC\left( R_{c} \right) \right)$ as a function of system parameters. It shows that for a Gaussian distribution of parameters, with mean as in Table 1 (main text) and standard deviation 1/30 of the mean, an increase in the recovery rate of humans and biting heterogeneity increase the threshold and therefore are beneficial for control.

### Two patch model $\boldsymbol{R}_{\boldsymbol{0}\boldsymbol{S}}$

The expression for basic reproduction number $R_{0S}$ in the spatialy heterogeneous model is given in the main text. Here the we expand it term by term and relate them to parameters of isolated patches.

|  | $\begin{matrix} \begin{matrix} \boldsymbol{R}_{\boldsymbol{0}\boldsymbol{S}}\boldsymbol{=}\sqrt{\frac{\boldsymbol{1}}{\boldsymbol{2}}\left( \boldsymbol{R}_{\boldsymbol{01}}^{\boldsymbol{2}}\boldsymbol{+}\boldsymbol{R}_{\boldsymbol{02}}^{\boldsymbol{2}}\boldsymbol{+}\boldsymbol{R}_{\boldsymbol{0}\boldsymbol{h}\boldsymbol{21}\boldsymbol{v}\boldsymbol{12}}^{\boldsymbol{2}}\boldsymbol{+}\boldsymbol{R}_{\boldsymbol{0}\boldsymbol{h}\boldsymbol{12}\boldsymbol{v}\boldsymbol{21}}^{\boldsymbol{2}}\boldsymbol{+}\boldsymbol{R}_{\boldsymbol{sqrt}} \right)} \\ \boldsymbol{R}_{\boldsymbol{sqrt}}\boldsymbol{=}\sqrt{{\boldsymbol{4}\boldsymbol{R}}_{\boldsymbol{01}}^{\boldsymbol{2}}\boldsymbol{R}_{\boldsymbol{02}}^{\boldsymbol{2}}\left( \boldsymbol{1-}\frac{\boldsymbol{p}_{\boldsymbol{12}}\boldsymbol{p}_{\boldsymbol{21}}}{\boldsymbol{p}_{\boldsymbol{11}}\boldsymbol{p}_{\boldsymbol{22}}} \right)\left( \frac{\boldsymbol{p}_{\boldsymbol{12}}\boldsymbol{p}_{\boldsymbol{21}}}{\boldsymbol{p}_{\boldsymbol{11}}\boldsymbol{p}_{\boldsymbol{22}}}\boldsymbol{-1} \right)\boldsymbol{+}\left( \boldsymbol{R}_{\boldsymbol{01}}^{\boldsymbol{2}}\boldsymbol{+}\boldsymbol{R}_{\boldsymbol{02}}^{\boldsymbol{2}}\boldsymbol{+}\boldsymbol{R}_{\boldsymbol{0}\boldsymbol{h}\boldsymbol{21}\boldsymbol{v}\boldsymbol{12}}^{\boldsymbol{2}}\boldsymbol{+}\boldsymbol{R}_{\boldsymbol{0}\boldsymbol{h}\boldsymbol{12}\boldsymbol{v}\boldsymbol{21}}^{\boldsymbol{2}} \right)^{\boldsymbol{2}}} \\ \boldsymbol{R}_{\boldsymbol{0}\boldsymbol{h}\boldsymbol{21}\boldsymbol{v}\boldsymbol{12}}\boldsymbol{=}\frac{\boldsymbol{N}_{\boldsymbol{02}}}{\boldsymbol{N}_{\boldsymbol{01}}}\left( \frac{\boldsymbol{b}^{\boldsymbol{2}}\boldsymbol{\beta}_{\boldsymbol{v}}^{\boldsymbol{2}}\boldsymbol{\beta}_{\boldsymbol{1}}^{\boldsymbol{2}}\boldsymbol{p}_{\boldsymbol{12}}^{\boldsymbol{2}}\boldsymbol{\Lambda}_{\boldsymbol{v}\boldsymbol{1}}\boldsymbol{\mu}_{\boldsymbol{H}\boldsymbol{2}}\boldsymbol{\sigma}_{\boldsymbol{v}\boldsymbol{1}}\left( \boldsymbol{\rho}_{\boldsymbol{1}}\boldsymbol{Q}_{\boldsymbol{22}}\boldsymbol{+}\boldsymbol{f}_{\boldsymbol{2}}\boldsymbol{\sigma}_{\boldsymbol{H}\boldsymbol{2}} \right)}{\boldsymbol{\mu}_{\boldsymbol{v}\boldsymbol{1}}^{\boldsymbol{2}}\boldsymbol{\Lambda}_{\boldsymbol{H}\boldsymbol{2}}\boldsymbol{Q}_{\boldsymbol{12}}\boldsymbol{Q}_{\boldsymbol{22}}\boldsymbol{Q}_{\boldsymbol{v}\boldsymbol{1}}} \right) \\ \boldsymbol{R}_{\boldsymbol{0}\boldsymbol{h}\boldsymbol{12}\boldsymbol{v}\boldsymbol{21}}\boldsymbol{=}\frac{\boldsymbol{N}_{\boldsymbol{01}}}{\boldsymbol{N}_{\boldsymbol{02}}}\left( \frac{\boldsymbol{b}^{\boldsymbol{2}}\boldsymbol{\beta}_{\boldsymbol{v}}^{\boldsymbol{2}}\boldsymbol{\beta}_{\boldsymbol{H}}^{\boldsymbol{2}}\boldsymbol{p}_{\boldsymbol{21}}^{\boldsymbol{2}}\boldsymbol{\Lambda}_{\boldsymbol{v}\boldsymbol{2}}\boldsymbol{\mu}_{\boldsymbol{H}\boldsymbol{1}}\boldsymbol{\sigma}_{\boldsymbol{v}\boldsymbol{2}}\left( \boldsymbol{\rho}_{\boldsymbol{1}}\boldsymbol{Q}_{\boldsymbol{21}}\boldsymbol{+}\boldsymbol{f}_{\boldsymbol{2}}\boldsymbol{\sigma}_{\boldsymbol{H}\boldsymbol{1}} \right)}{\boldsymbol{\mu}_{\boldsymbol{v}\boldsymbol{2}}^{\boldsymbol{2}}\boldsymbol{\Lambda}_{\boldsymbol{H}\boldsymbol{1}}\boldsymbol{Q}_{\boldsymbol{11}}\boldsymbol{Q}_{\boldsymbol{21}}\boldsymbol{Q}_{\boldsymbol{v}\boldsymbol{2}}} \right) \end{matrix} \end{matrix}$ | (8) |
| --- | --- | --- |

1. Keeling MJ and Rohani P. *Modeling infectious diseases in humans and animals*: Princeton University Press, 2008.

2. Anderson RM, May RM and Anderson B. *Infectious diseases of humans: dynamics and control*: Wiley Online Library, 1992.

3. Van den Driessche P and Watmough J. Reproduction numbers and sub-threshold endemic equilibria for compartmental models of disease transmission. Math Biosci 2002.

4. Kong L, Wang J, Li Z, et al. Modeling the heterogeneity of Dengue transmission in a city. International journal of environmental research and public health 2018.

5. Kong L, Wang J, Han W, et al. Modeling heterogeneity in direct infectious disease transmission in a compartmental model. International journal of environmental research and public health 2016.

6. Vynnycky E and White R. *An introduction to infectious disease modelling*: OUP oxford, 2010.

7. McCallum H, Barlow N and Hone J. How should pathogen transmission be modelled?. Trends in ecology & evolution 2001.

8. Jiang J, Qiu Z, Wu J, et al. Threshold conditions for West Nile virus outbreaks. Bull Math Biol 2009.

9. Gumel AB. Causes of backward bifurcations in some epidemiological models. Journal of Mathematical Analysis and Applications 2012.

10. Mitra C, Kurths J and Donner RV. An integrative quantifier of multistability in complex systems based on ecological resilience. Scientific reports 2015.

11. Marino S, Hogue IB, Ray CJ, et al. A methodology for performing global uncertainty and sensitivity analysis in systems biology. J Theor Biol 2008.
